# Supplementary material for: Amino Acid Digestibility of Different Formulations of Torula Yeast in an In Vitro Porcine Gastrointestinal Digestion Model and Their Protective Effects on Barrier Function and Inflammation in a Caco-2/THP1Co-Culture Model
Source: Animals (Basel). 2023 Sep 5;13(18):2812. doi: 10.3390/ani13182812 (PMC10526019; doi:10.3390/ani13182812)
Supplement: Supplementary file 1 [file animals-13-02812-s001.zip › animals-2473169-supplementary.pdf]

Supplementary information

Table S1. Mobile phase gradient used in amino acid separation by HPLC-DAD.

Time (min)	*%Solvent A*	*%Solvent B*	*%Solvent C*
0	*7,8*	*87,0*	*5,2*
11	*11,1*	*81,6*	*7,4*
21	*19,2*	*68,0*	*12,8*
41	*60,0*	*0,0*	*40,0*
41,5	*7,8*	*87,0*	*5,2*
51	*7,8*	*87,0*	*5,2*
